# How do vibration stimulation frequencies affect the nonlinear dynamics and mechanical characterization of breast cancer cells?

**DOI:** 10.1016/j.bbrep.2025.102414

**Published:** 2025-12-15

**Authors:** Ashkan Heydarian, Dornaz Milani, Hamidreza Mortazavy Beni, Mehrafarin Babaee, Hamid Reza Goudarzi

**Affiliations:** a Neuromusculoskeletal Rehabilitation Research Center, University of Social Welfare and Rehabilitation Sciences, Tehran, Iran; b Department of Biomedical Engineering, Science and Research Branch, Islamic Azad University, Tehran, Iran; c Department of Biomedical Engineering, Ars. C., Islamic Azad University, Arsanjan, Iran; d Mathematics Department, Yasouj University, Yasouj, Iran

**Keywords:** Breast cancer, Cell mechanics, FEM, Mechanical frequencies, Largest Lyapunov exponent

## Abstract

**Introduction:**

Understanding the mechanical properties of cells is crucial for gaining insights into their physiological and pathological states. This study focuses on the mechanical behavior of human mammary epithelial cells (MCF-10) and human breast cancer cells (MCF-7), emphasizing mechanical frequencies, finite element modeling (FEM), and nonlinear dynamics of the cells.

**Methods:**

Cells were cultured and subjected to mechanical testing using Atomic Force Microscopy (AFM) and Magnetic Tweezer Cytometry (MTC). The elastic and viscoelastic properties were analyzed, and FEMs were developed to simulate cell behavior under various mechanical stimuli. The nonlinear dynamic behavior was examined using the Duffing model, and chaos was assessed using the Largest Lyapunov exponent (LLE).

**Results:**

MCF-10 cells exhibited higher stiffness than MCF-7 cells. The mechanical frequencies of both cell types were determined, and significant differences were observed at higher frequencies. FEM simulations provided detailed insights into the stress distribution and deformation patterns within cells. The nonlinear analysis revealed chaotic behavior at specific frequencies, particularly in the range of 22–36 kHz.

**Conclusion:**

Identifying the mechanical frequencies and responses of cancer cells, including their nonlinear and chaotic behaviors, can inform the development of noninvasive therapeutic strategies. Further research is required to refine these models and explore the potential of mechanical forces in cancer treatment**.**

## Highlights

• Investigated mechanical properties of MCF-10 and MCF-7 cells using AFM and MTC.
• Analyzed nonlinear dynamics and chaos in cell mechanics using the Duffing model and Largest Lyapunov exponent.
• FEM simulations revealed stress distribution and deformation patterns in epithelial and cancer cells.
• Identified chaotic behavior in cancer cells at mechanical frequencies (22–36 kHz).
• Findings suggest potential applications of mechanical forces in non-invasive cancer therapies.

## 1 Introduction

Cells are the fundamental structural and functional units of all living organisms. These living cells, regarded as intricate and dynamic entities, exhibit a multitude of structural and physical properties that enable them to respond to various physiological conditions and mechanical stimuli. This responsiveness occurs both within the organism and in its external environment. Any deviations from these established properties not only compromise the physical integrity and stability of cellular structures but also threaten their essential biological function. Such deviations can lead to a range of deleterious effects on homeostasis, affecting the entire organism [1,2].

Cancer, one of the most significant health challenges of our time, arises from uncontrolled and aberrant proliferation of cells, leading to the formation of malignant tumors. This inappropriate cell growth not only invades the surrounding healthy tissues but also causes substantial damage, leading to various complex disorders. These disorders are classified based on the specific cell type involved or the tissue from which the malignancy originates [3,4]. International statistical data on oncological diseases reveal that breast cancer (BC) is alarmingly prevalent, with approximately 2.8 million cases worldwide. This makes BC one of the most common malignant tumors among women and the second leading cause of cancer-related deaths in females worldwide. Moreover, the incidence of breast cancer is rising in various regions worldwide [5]. This increase can be partly attributed to advancements in diagnostic techniques and the heightened prevalence of risk factors, particularly in developing nations. A wide array of cancer therapies, including tumor excision, chemotherapy, and hormone-based treatments are employed worldwide. However, these treatments often face limitations and challenges that can significantly affect their overall efficacy and patient outcomes [6,7].

Researchers are exploring the potential use of mechanical waves for non-invasive and effective cancer treatment. These methods can be broadly classified into thermal and nonthermal categories based on their physiological impact. The frequency and intensity of mechanical waves play crucial roles in these treatments. Numerous studies have examined the effects of audible acoustics and ultrasound (US) waves on cellular and tissue structures [8,9]. Non-thermal ultrasound, characterized by its ability to induce minimal temperature changes within the treated tissues, can be classified into two main mechanisms: sonomechanical and sonochemical. Sonomechanical effects, driven by cavitation and acoustic forces, generate significant shearing forces, shock waves, and microjets, leading to damage to cellular membranes. These forces can influence cellular proliferation, protein synthesis, cytoskeletal rearrangement, and gene transfection [10,11].

The sonochemical effects primarily result from the generation of reactive oxygen species (ROS) through inertial cavitation. ROS can trigger harmful biological responses, such as oxidative stress, mitochondrial dysfunction, DNA damage, and apoptosis in cancer cells. The synthesis and accumulation of ROS can be enhanced using sonosensitizers or drugs through a process known as sonodynamic therapy (SDT). SDT is a minimally invasive approach used to treat solid malignant tumors by administering a sensitizing drug, applying ultrasound waves, and ensuring the presence of molecular oxygen. In oncology, SDT is often compared to photodynamic therapy (PDT); however, ultrasound has the advantage of better tissue penetration and precise tumor targeting [[12], [13], [14]].

Ultrasound methodologies, which rely on parameters such as sound wave frequency and intensity, can be classified into High-Intensity Focused Ultrasound (HIFU) and Low-Intensity Pulsed Ultrasound (LIPUS). HIFU, with frequencies between 0.8 and 5 MHz, is used for cancer treatment through thermal and non-thermal mechanisms, such as hyperthermia and acoustic radiation force [15]. HIFU is non-invasive, preserves the integrity of the surrounding tissue, and shows potential as an alternative or complementary treatment for breast cancer management [16,17].

LIPUS uses low-energy ultrasound waves to stimulate cell proliferation, differentiation, and matrix synthesis, and has applications in wound healing, bone fracture repair, and drug delivery. Advanced methodologies include Ultrasound-Mediated Gene Delivery (UMGD), which enhances gene vector transfection into cells or tissues, and Ultrasound Reversal of Multidrug Resistance (MDR), which increases cancer cell sensitivity to treatment by modulating transporter proteins [18,19].

Focused ultrasound waves concentrate energy on specific tissue areas, significantly raising the temperature and causing coagulative necrosis in the tissue. When combined with the chemotherapeutic agent paclitaxel, 1.5 MHz HIFU markedly enhanced therapeutic efficacy [20]. Hyperthermia magnetic devices using engineered nanoparticles have been employed in breast cancer treatment to reduce tumor size and potentially augment the effectiveness of radiotherapy. These devices operate between 108 and 360 kHz, with the best performance at 330 kHz. Research on the impact of magnetic hyperthermia on breast cancer cells in mice at 250–350 kHz shows promising outcomes [21,22]. Studies from previous decades have shown that 1 MHz waves can alter cancer cell morphology. Focused ultrasound transducers or intensities above 50 MHz disrupt cancer cells [23,24]. Mechanical investigations of cancer cells and their biophysical properties can significantly contribute to the development of new diagnostic and therapeutic strategies [25]. The transformation of healthy cells into cancerous cells involves multiple biophysical changes. Healthy breast cells have natural frequencies between 22,000 and 36,000 Hz, whereas breast cancer cells have frequencies ranging from 9000 to 16,000 Hz. Changes in cell adhesion mechanisms significantly impact these frequencies [26].

Cancer metastasis is linked to systemic breakdowns in subsystems. The unpredictable evolution of cancer cells leads to chaotic systems with dynamic behaviors, complicating treatment [27,28]. Many cancers exhibit spatiotemporal heterogeneity, which can be described using chaos theory [29]. Tumors, as dynamic nonlinear systems, require nonlinear analyses for stability and behavioral assessments over time [30]. Studying cancer cell dynamics as chaotic systems can aid in targeted therapy. The natural frequencies of healthy cells differ from those of cancerous cells [31]. Chaos in biological systems can be quantified using the Lyapunov exponent, which measures cellular system dynamics [32].

Given the complexity of biological materials, linear mechanical models are often inadequate. Nonlinear models offer more accurate representation. Using a nonlinear framework, we examined the mechanical responses of healthy and cancerous breast epithelial cells to mechanical wave exposure. Finite element analysis was used to calculate the natural frequencies of these cell groups [33,34].

This study aimed to comprehensively investigate the mechanical properties and behavior of (MCF-10 and MCF-7) when subjected to various mechanical stimuli. This study sought to determine the mechanical frequencies of these cell types and analyze their elastic and viscoelastic properties using advanced techniques, such as AFM and MTC. Additionally, FEM will be developed to simulate the behavior of cells under mechanical stress, providing detailed insights into stress distribution and deformation patterns. This study also aims to explore the nonlinear dynamic behavior of the cells through the application of the Duffing model and assess chaotic behavior using the Lyapunov exponent, particularly at specific frequency ranges. By achieving these objectives, this study aims to enhance our understanding of the mechanical responses of cancer cells, which could inform the development of innovative, non-invasive therapeutic strategies. Here, we introduce an integrated, mechanics-forward framework that couples single-cell force spectroscopy (AFM) and magnetic twisting cytometry with high-fidelity finite element modeling and nonlinear systems analysis to resolve how healthy (MCF-10) and malignant (MCF-7) breast epithelial cells store, dissipate, and dynamically amplify mechanical energy. By jointly extracting elastic–viscoelastic parameters, computing natural frequencies from physiological to kilohertz bands, and fitting a Duffing oscillator while quantifying Lyapunov exponents, we delineated frequency-selective regimes, including chaotic transitions, where phenotypic contrasts were maximized. This fusion of measurement and modeling elevates cell mechanics from descriptive stiffness readouts to predictive frequency-domain “vulnerability maps” that can guide non-thermal ultrasound designs and other sonomechanical interventions. Beyond enabling robust mechanobiological biomarkers for discrimination, our pipeline yields actionable bounds on excitation amplitude and frequency compatible with noninvasive delivery, laying a quantitative foundation for precision mechanics-guided cancer therapeutics and rational optimization of sonodynamic or combinatorial strategies.

## 2 Material and methods

### 2.1 Cell culture

Two human mammary cell lines, MCF-10A and MCF-7, were kept at 37 °C in a humidified 5 % CO2 atmosphere in Dulbecco's Modified Eagle Medium (DMEM; Gibco, Thermo Fisher Scientific) supplemented with 20 % fetal calf serum (FCS; Sigma-Aldrich). Cells were plated at the same seeding densities and kept within the same passage range across cell types; when used, cultures were grown in 35 mm Petri dishes (12 dishes per experiment when specified), and the cell condition (density and morphology) was checked by light microscopy prior to mechanical testing. Primary mechanical measurements (AFM, magnetic twisting cytometry [MTC], sinusoidal, and creep assays) used identical DMEM + 20 % FCS conditions for both cell lines unless explicitly stated otherwise.

N-Methyl-2-pyrrolidone (NMP; Merck) particles were prepared from a 1000 ppm stock to obtain a 7 % (v/v) working solution (final concentration in culture medium) and functionalized with folic acid (NanoZino). The NMP particle preparation had a particle size distribution centered at ∼1065 nm. NMP served two roles: (1) calibration of the MTC force using capillary calibration tubes, and (2) a limited exploratory pre-treatment arm in which cultures were incubated with folate-functionalized NMP for 12 h prior to mechanical testing; control plates for AFM and MTC measurements were cultured without NMP. The reported mechanical data compare cells maintained in identical baseline culture conditions, except where the separate NMP pre-treatment arm is explicitly stated.

Six Petri dishes were sent to a laboratory equipped with an AFM for analysis, and another six were sent to a laboratory equipped with an MTC. Throughout the process, the temperature of the Petri dishes was maintained at 37*°C*.

### 2.2 Elastic behavior by AFM

AFM (JPK, Germany) was used to investigate the elastic behavior of the two cell lines. The device used was an AppNano quadrilateral pyramid (model HYDRA6V–200 N) made of silicon nitride with a spring constant of 0.045 N/m. Prior to testing, the device was sterilized and calibrated using ultraviolet radiation, according to the sterilization protocol.

For a pyramidal indenter with half-angle α, the force–indentation relation was fitted using the appropriate Hertz-like relation for pyramidal probes. In our analysis, we used the widely adopted form.

A contact force was applied at 20 points per cell, and force indentation curves were acquired from 50 individual cells per group. Deformation curves were recorded by plotting the force-indentation data. The force-indentation data from the extended portion of the AFM results were analyzed for both the cell lines. Finally, the average data for each group were calculated. The entire process of testing and data extraction was completed within 2 h at a chamber temperature of 37 °C.

In this study, the cell stiffness was measured to determine the Young's modulus of the cell samples. Young's modulus was derived from the deformation-force curves of MCF-7 and MCF-10 cells, and the results were compared using the Hertz model (Eq. (1)).

(1) $$E=\frac{\surd 2\;\left(1-\nu^{ˆ}2\;\right)F}{\;\varphi^{ˆ}2\;\tan\theta }$$

In this study, quadrilateral probe **E** represents the Young's modulus of the cell. The parameters used included the half-angle to the face of the probe Ɵ at 45°, Poisson's ratio **ν** for biological samples set at 0.5, deformation depth **φ**, and the force applied to the cell **F**. The Young's modulus for each cell was calculated, and the results for the two cell lines were averaged. Each cell line was tested using 50 cells. Statistical analysis was conducted using Minitab software. The normality of the Young's modulus data for each group was assessed using the Anderson-Darling test.

### 2.3 Sinusoidal test using MTC

After culturing, the cells were subjected to a force of 299 *pN* using the MTC device. This device, equipped with a coil featuring 1500 turns of 0.7 mm diameter copper wire generating a 3*A* current, was calibrated using a capillary tube containing NMP. The magnetic force was calibrated using a capillary tube filled with functionalized NMP particles. The coil current–force relationship was measured prior to each experiment. After calibration, the magnetic field strength and applied force analogies to NMP were measured [35]. The cells were preconditioned with 10 cycles of loading/unloading at 0.5 Hz and 300 pN amplitude. This protocol was selected to minimize the history-dependent viscoelastic drift and obtain repeatable displacement traces. Cell motility was observed using a 5-megapixel camera attached to a microscope at 1000x magnification. Changes in cell morphology were tracked using a program that labeled cell membranes with 10 membrane markers to detect alterations in cell shape.

A sinusoidal force with a frequency of 0.5 Hz and amplitude of 300 *pN* was applied to 100 cells from each cell line. Membrane markers (10 per cell) were tracked using Tracker v6.0.2 with sub-pixel interpolation enabled. The displacements were calibrated using an image of a Neubauer chamber at the same magnification. This software determined the deformation of the cell membrane in a magnetic field by applying the marker technique across various frames. The software output provided movement data. Displacement-time data were collected for both cell lines, and the distances were calibrated using a Neubauer chamber. The test was conducted three times for each cell to ensure data accuracy. In addition, the displacement of ten different membrane locations per cell was measured. According to the Anderson-Darling test, the data for each cell were normally distributed. Cell selection was random, and the sample size was estimated using a pilot test.

The nonlinear data obtained from the evaluation were meticulously analyzed using Python software (version 3.12), and the Duffing model was subsequently fitted to the data. The Duffing equation is renowned for its capacity to exhibit a wide spectrum of dynamic behaviors, including periodic, quasi-periodic, and chaotic motion, each highly dependent on the specific values of the system parameters. This well-known nonlinear second-order differential equation is instrumental in modeling the dynamics of certain damped and driven oscillators (Eq. (2)).

(2) $$\ddot{x}+\delta \dot{x}+\alpha x+\beta x^{3}=\gamma \;\sin\left(\omega t\right)$$

Where **x** is the displacement, $\dot{x}$ is the velocity (first derivative of **x** with respect to time), $\ddot{x}$ is the acceleration (second derivative of **x** with respect to time), δ is the damping coefficient, α and β are constants that define the linear and nonlinear stiffness, respectively. Also, γ is the amplitude of the periodic driving force, ω is the angular frequency of the periodic driving force.

The term $\delta \dot{x}$ has been represented as the damping force, which has opposed the motion and reduced the energy over time. The terms αx and $\beta x^{3}$ have represented the restoring force. The αx term has been linear, while the $\beta x^{3}$ term has introduced non-linearity. The term γsin⁡(ωt) represents an external periodic driving force that has added energy to the system. The Lyapunov exponent was measured to assess the stability against frequency. If all responses are positioned around the equilibrium point by setting the initial state near this point, it indicates stability with respect to frequency.

We computed the maximum Lyapunov exponent using the Rosenstein method implemented on displacement time series after detrending and phase-space embedding (embedding dimension m = 6; delay τ chosen from the first minimum of the mutual information). The reported LLE is the slope of the mean logarithmic divergence curve over the selected linear region.

After fitting the model to the experimental data and obtaining the coefficients, the nonlinear differential equation was solved numerically using Python. The Lyapunov exponent was evaluated at different frequencies. This exponent, which is one of the key indicators in the dynamic system, suggests that the system exhibits chaotic behavior. The Lyapunov exponent is a key measure in dynamic systems that is used to identify chaos. This indicates how quickly the nearby trajectories diverge over time. A positive Lyapunov exponent indicates that small initial differences grow exponentially, leading to chaotic behavior. This exponent is calculated by iterating the system equations and observing the trajectory divergence. This provides insights into the stability and predictability of system. A positive Lyapunov exponent is a hallmark of chaos, indicating the system's sensitivity to initial conditions and resulting in complex, unpredictable dynamics.

### 2.4 Dynamic creep test by MTC

Subsequently, a dynamic creep test was conducted on an additional 100 cells from each cell line under the applied loading conditions, following the principles of integral heritage. The stair loading force was applied in four stages, with each stage involving a force increment of 300 pN at a constant frequency of 0.5 Hz, utilizing the MTC device [36].

The creep dynamic test was conducted by applying a load that adhered to the principles of hereditary integral, which was specifically generated for the two cell lines. This approach aimed to identify the most suitable dynamic viscoelastic model based on experimental data. The best model for the viscoelastic properties of the target cells was determined by minimizing the residual error using artificial intelligence algorithms in the Python software. Based on the data, the four-element viscoelastic fluid model was chosen, and the corresponding equation was formulated as Eq. (3):

(3) $$\varepsilon \left(t\right)=\int_{0}^{t}J\left(t-\tau \right)\sigma \left(\tau \right)\;d\tau$$

where**ε(t)** is the strain at time **t**, **J (t**–**τ)** is the compliance function, which describes the material's response at time **t** due to a stress applied at an earlier time **τ**, and **σ (τ)** is the stress applied at time **τ**.

This integral equation shows how the strain at any given time depends on the entire history of the applied stress and the compliance of the material.

In practical terms, if you have a constant applied stress **σ_0_**, the strain **ε(t)** can be simplified to Eq. (4):

(4) $$\varepsilon \left(t\right)=\sigma_{0}.\;J\left(t\right)$$

where **J(t)** is the compliance function we discussed earlier, described as Eq. (5):

(5) $$J\left(t\right)=J_{0}+J_{1}\left(1-e^{-\frac{t}{\tau_{1}}}\right)+J_{2}\left(1-e^{-\frac{t}{\tau_{2}}}\;\right)$$

This relationship shows how the material's strain evolves over time under a constant applied stress. **J(t)** is the compliance at time **t**, **J_0_** is the instantaneous compliance, **J_1_** and **J_2_** are the retardation compliances, **τ_1_** and **τ_2_** are the retardation times. The viscoelastic properties of both cell lines were extracted.

### 2.5 Finite element simulation

**Fig. 1:**
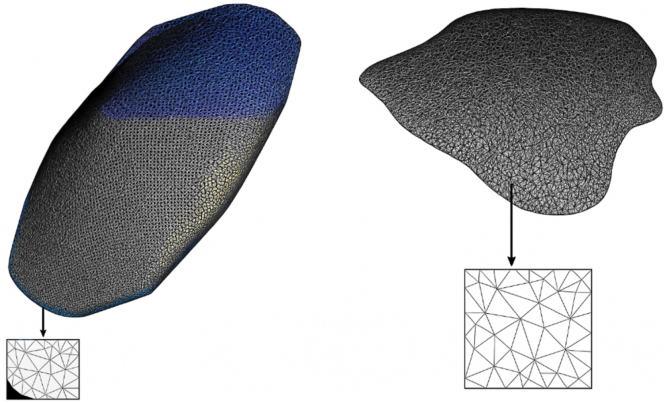
3D modeling of A. MCF-10 and B. MCF-7 cells using AI algorithms in python.

Artificial intelligence algorithms in Python software, along with microscopic images of two-dimensional MCF-10 and MCF-7 cells, were utilized to generate three-dimensional models [37]. These 3D models of both cell types were then imported into the commercial finite element package ABAQUS (standard version 6.13, SIMULIA, Germany) for further analysis (Fig. 1).

Two simulations were performed using elastic and viscoelastic stiffness matrices. In the first simulation, a rigid body model was selected, and the mechanical properties of the cells were assumed to be elastic. The properties of homogeneous, isotropic, and elastic materials were considered for the two-cell model. The Young's modulus of both cell groups, obtained during the laboratory investigation of their elastic properties, was based on prior research, with a Poisson's ratio set at 0.5. The simulation aimed to replicate the mechanical behavior of the cells under various conditions. Boundary conditions and loading scenarios were applied to mimic the experimental setup accurately, with the same cell attached to the Petri dish substrate from the bottom side in all directions.

In the subsequent step, both cell models were subjected to meshing. The MCF-10 cell meshes were tetrahedral elements (C3D10), comprising 560,926 elements, while the MCF-7 cell meshes also consisted of tetrahedral elements (C3D10) comprising 640,282 elements.

For the viscoelastic simulation, the homogeneous, isotropic, and viscoelastic material properties were assumed for the two cell models. The Prony series for time-domain viscoelasticity was chosen in ABAQUS. The parameters **J_1_** and **τ_1_** for the first term, and **J_2_** and **τ_2_** for the second term were entered. The instantaneous compliance **J_0_** was defined in the Elastic section as the inverse of the instantaneous modulus **E_0_**. Based on prior research, with Poisson's ratio set at 0.5, boundary conditions and loading scenarios were applied to mimic the experimental setup.

In the subsequent step, both cell models were subjected to meshing, and a mesh convergence test was conducted to determine the optimal mesh refinement size. Meshing was performed similarly to the elastic model.

To ensure the validity of the FEM results, a force similar to that used in Elastic Behavior by AFM was applied to the MCF-7 cell.

The detailed characteristics of both cell types are presented in Table 1, organized based on the modeling parameters of each cell group. Subsequently, the natural frequencies of both cell types were determined using Abaqus software.

**Fig. 2:**
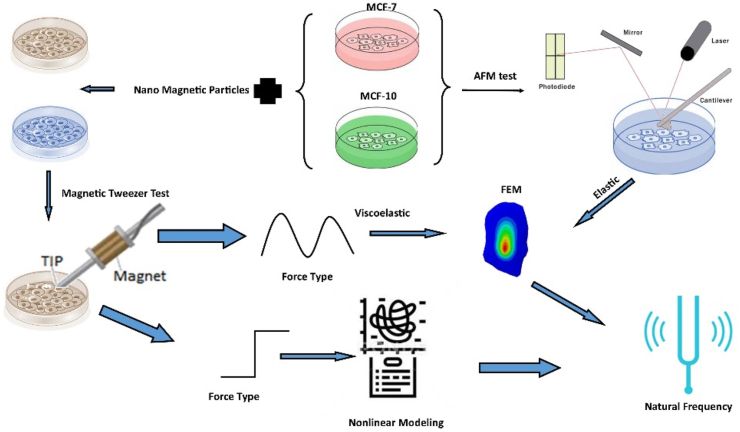
Schematic workflow for mechanical characterization and modeling of MCF-7 and MCF-10 cells. Mechanical testing was performed using Atomic Force Microscopy (AFM) and Magnetic Tweezer Cytometry (MTC), with force profiles classified as elastic or viscoelastic. Elastic and viscoelastic data were used to inform finite element models (FEM), and nonlinear modeling was applied to assess dynamic behavior and natural frequency estimation.

All frequency analysis tests whether conducted in nonlinear mode to detect chaos or using finite element methods to determine the cell's natural frequency within the 0–200 Hz range, accounted for the natural vibrations of the environment. All experiments and approaches were depicted in schematic Figure (2).

## 3 Results

### 3.1 AFM result

In calculating the elastic behavior of MCF-7 and MCF-10 cells using deformation force curves, the average Young's modulus was determined to be 724.35 N/m^2^ for 20 points across 50 MCF-10 cells, and 251.23 N/m^2^ for 50 MCF-7 cells. These measurements were used for FEM modeling of the elastic properties of both cell lines.

### 3.2 Dynamic creep test

For the viscoelastic behavior, a dynamic creep test was conducted by applying heredity integral, specifically generated for the two cell lines. Based on the data, the four-element viscoelastic fluid model was chosen. The parameters obtained from the curve fitting for both the MCF-10 and MCF-7 cell lines are presented as follows. For MCF-10 cells, the retardation compliances J_1_and J_2_ were 0.0065 m^2^*/pN* and 4.5579 $\times 10^{-7}$ *m*^*2*^*/pN*, respectively. The retardation times t_1_ and t_2_ were 1.4557 s and 3.0806 s, respectively. The instantaneous Modulus E_0_ was 774.2965 N/m^2^*,* with a Root Mean Square Error (RMSE) of 0.0004.

**Fig. 3:**
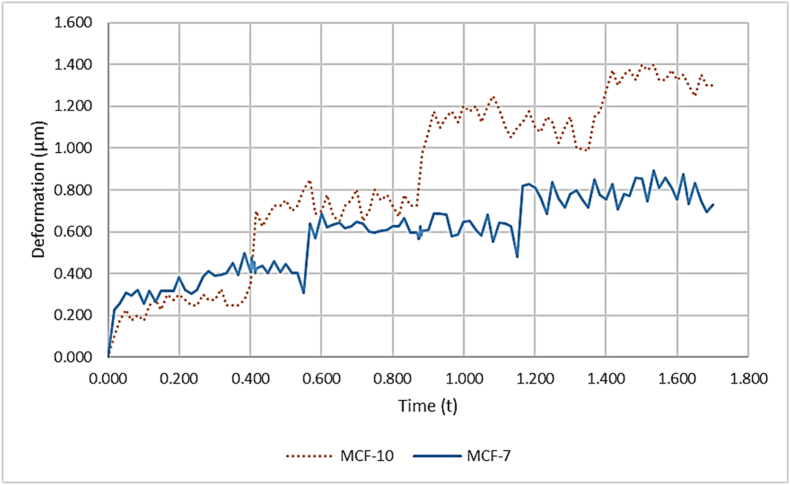
The dynamic creep response: Deformation over time for (MCF-10 and MCF-7) Cell Lines.

For MCF-7 cells, the parameters J_1_ and J_2_ were both 0.0008 m^2^*/pN.* The retardation times τ_1_ and τ_2_ were 0.0086 *s* and 2.1287 *s*, respectively. The instantaneous Modulus E_0_ was 283.9029, with a Root Mean Square Error (RMSE) of 0.0002 (Fig. 3). These measurements were utilized for FEM modeling of the viscoelastic properties of both cell lines.

Fig. 3 shows the creep response of two breast epithelial cell lines under a multi-step load. Each step produces an immediate deformation jump (elastic component) followed by a gradual rise toward a quasi-plateau (viscoelastic creep). The dotted brown trace (MCF-10) consistently attains larger absolute deformations and approaches its plateaus faster; by the end of the protocol, it reaches ≈1.3–1.4 μm. The solid blue trace (MCF-7) saturates lower, at ≈0.8–0.9 μm, under the same loading. Thus, MCF-10 exhibits higher compliance (roughly 50–70 % greater steady-state deformation) indicating lower effective stiffness and more pronounced creep. The small fluctuations, especially near step transitions, reflect transient dynamics and cytoskeletal rearrangement rather than noise. Overall, the figure highlights a clear mechanical phenotype gap: MCF-10 behaves softer and more load-sensitive, whereas MCF-7 maintains greater resistance information that can be leveraged for mechanical discrimination and for tuning amplitude/timing in non-thermal sonomechanical stimulation.

### 3.3 Finite element analysis results

According to the modeling, the MCF-10 cell has an area of 298 μm^2^ and reaches a height of 4.5 μm at its highest point, while the MCF-7 cell has an area of 2272 μm^2^ and reaches a height of 3.2 μm at its highest point. In the study of elastic behavior, properties were considered with a density of 1000 kg/m^3^ and Poisson's ratio of 0.5.

**Fig. 4:**
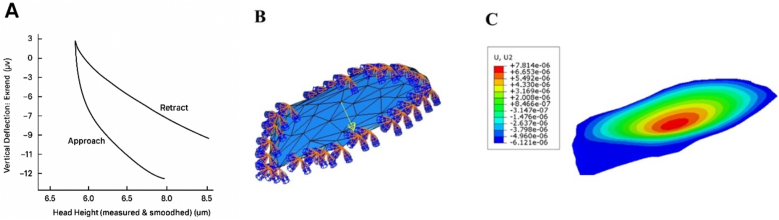
(A) A force similar to that used in Elastic Behavior by AFM was applied to the MCF-7 cell. (B) Diagram of the force displacement applied to the MCF-7 cell (C) Applying force in MCF-7 Elastic behavior.

The results of the FEM study, in which a contact force ranging from 3 to 12 pN was applied to the MCF-7 cell for 1 s to observe the elastic behavior using AFM, are as follows (Fig. 4). This figure integrates three complementary stages of the methodology:

A – Force–Indentation Curve (AFM):

The left panel shows the typical approach and retracts branches recorded during an AFM indentation test. The hysteresis between loading and unloading reflects viscoelastic behavior and energy dissipation within the cell. These data are used to extract elastic and viscoelastic parameters such as the Young's modulus.

B – Finite Element Mesh Model:

The middle panel presents the meshed geometry of a reconstructed cell, discretized into triangular elements for finite element analysis. This mesh enables numerical simulation of stress and strain distributions under applied mechanical loads. The refined mesh along curved regions ensures accurate resolution of deformation patterns.

C – Displacement Contour Map:

The right panel illustrates the finite element results as a colored displacement field. The contour plot (U2) highlights regions of maximum deformation in warm colors (red–yellow) and areas of minimal displacement in cool colors (blue green). This spatial distribution identifies zones of mechanical vulnerability and indicates how the cell redistributes stresses.

Taken together, panels A–C demonstrate the experimental-to-computational pipeline: AFM provides direct mechanical input, meshing builds a numerical framework, and finite element simulations yield detailed deformation maps. This integrative approach deepens the understanding of single-cell mechanics and supports the comparison of healthy versus cancerous phenotypes.

Mesh convergence results are shown in Fig. 6 Both MCF 10A and MCF 7 curve plateau beyond ≈50,000 elements, indicating numerical convergence. Consequently, the computed first natural frequency near 1.4 Hz is mesh independent and differences between cell types are driven by geometry and material parameters rather than discretization artifacts. We therefore adopted meshes with ≥100,000 elements for all modal analyses to ensure a conservative margin for convergence (Fig. 6).

**Fig. 5:**
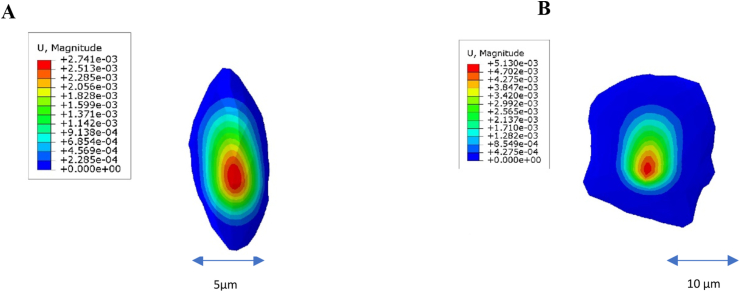
(A) Natural frequencies of MCF-10 Elastic behavior in 0–20 Hz Frequency range: 1.4216 Hz, (B) Natural frequencies MCF-7 Viscoelastic behavior in 0–20 Hz Frequency range: 1.7236 Hz.

The average values of Young's modulus obtained at each step in the frequency ranges are presented in Table 1. For the study of viscoelastic behavior, the density and Poisson's ratio were similarly considered, along with the elements obtained from the Prony series for each cell line, for the natural frequency ranges presented in Table 1. No resonant frequencies were detected within several specified intervals (Fig. 5). The figure presents finite element simulations of displacement magnitude for two cell morphologies under mechanical loading.

A (5 μm scale): The elongated, oval-shaped cell exhibits highly localized displacement at its central region. The deformation is concentrated, with peak displacements around 2.7 × 10^−3^ μm, indicating that stress is absorbed and confined primarily within the core area of the cell. This suggests a more localized and rigid mechanical response.
B (10 μm scale): The irregular, wider cell shows a broader distribution of displacement across its surface. Although the peak displacement is comparable (∼2.5–3.0 × 10^−3^ μm), the deformation is more diffused, implying greater compliance and a lower effective stiffness.

**Table 1:** Natural frequencies of MCF-10 and MCF-7 of Elastic and Viscoelastic behavior.

Frequency range	Natural frequencies of MCF-10 Elastic behavior	Natural frequencies MCF-7 Elastic behavior	Natural frequencies of MCF-10 Viscoelastic behavior	Natural frequencies MCF-7 Viscoelastic behavior
(0–20) Hz	1.4216 Hz	1.5766 Hz	1.4786 Hz	1.7236 Hz
(20–40) Hz	20.983 Hz	20.984 Hz	20.982 Hz	20.983 Hz
(40–60) Hz	40.970 Hz	41.004 Hz	40.961 Hz	41.070 Hz
(60–80) Hz	60.968 Hz	60.941 Hz	60.940 Hz	60.980 Hz
(80–100) Hz	81.130 Hz	81.100 Hz	80.959 Hz	80.932 Hz
(100–120) Hz	101.76 Hz	101.01 Hz	101.15 Hz	100.93 Hz
(120–150) Hz	121.29 Hz	121.05 Hz	121.17 Hz	121.25 Hz
(150–200) Hz	151.2 Hz	150.90 Hz	151.1 Hz	150.90 Hz
(9–16) kHz	9.820 kHz	9.0058 kHz	9.821 kHz	9.0758 kHz
(22–36) kHz	25.5713 kHz	–	26.5961 kHz	–
(108–360) kHz	344.6562 kHz	–	358.4764 kHz	–
(0.8–5) MHz	0.8938 MHz	–	0.9297 MHz	–

**Table 2:** Largest Lyapunov Exponent (LLE) for each frequency interval. 'Frequency' denotes the excitation frequency at which the maximum LLE within the stated interval was observed.

Frequency range	LLE of MCF-10	Frequency	LLE of MCF-7	Frequency
(0–20) Hz	0.0088	18.97436 Hz	0.0624	5 Hz
(20–40) Hz	0.0044	31.7949 Hz	0.0060	40.0 Hz
(40–60) Hz	0.0024	52.3077 Hz	0.0060	40.0 Hz
(60–80) Hz	0.0026	62.0513 Hz	0.0021	80.0 Hz
(80–100) Hz	0.0011	89.7436 Hz	0.0022	80.0 Hz
(100–120) Hz	0.0021	109.7436 Hz	0.0022	106.0759 Hz
(120–150) Hz	0.0020	139.3220 Hz	0.0083	130.5882 Hz
(150–200) Hz	0.0111	172.7273 Hz	0.0641	159.5960 Hz
(9–16) kHz	–	–	–	–
(22–36) kHz	2.0782	29.0268 kHz	1.3264	30.3996 kHz
(108–360) kHz	1.2564	242.1473 kHz	1.2050	358.5383 kHz
(0.8–5) MHz	1.5735	1.8848 MHz	1.1392	2.7223 MHz

**Fig. 6:**
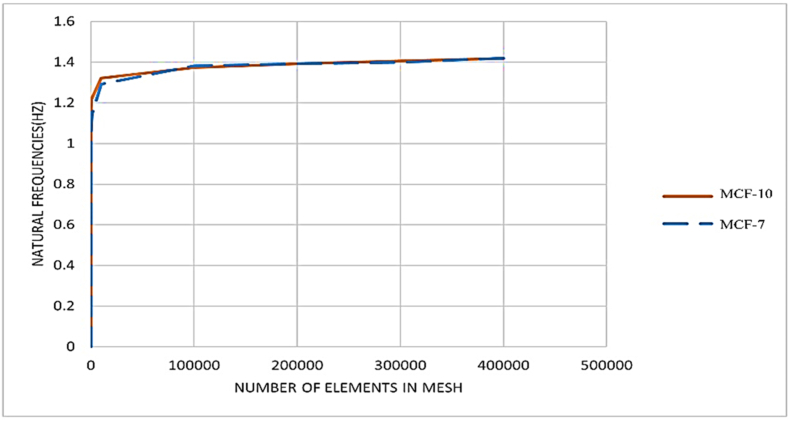
The mesh convergence test, conducted to determine the optimal mesh refinement size in elastic behavior at frequencies ranging from 0 to 20 Hz.

The results highlight structural and biomechanical differences: the oval-shaped cell demonstrates localized resistance, while the wider cell displays broader, less rigid deformation. Such variations in mechanical response can serve as indicators of distinct cellular phenotypes (e.g., normal vs. cancerous) and may guide sonomechanical or biomechanical characterization approaches.

Displays the results of the mesh convergence test, conducted to determine the optimal mesh refinement size in elastic behavior at frequencies ranging from 0 to 20 Hz. Fig. 6 illustrates the relationship between natural frequency (Hz) and the number of elements in the finite element mesh for two cell types, MCF-10 (normal epithelial cells) and MCF-7 (cancer cells). Both curves show a rapid increase in natural frequency as the number of mesh elements increases from very low values. After approximately 50,000 elements, the frequency values stabilize and asymptotically approach a plateau. This indicates mesh convergence, meaning that further refinement of the mesh has little effect on the computed frequency. The natural frequencies for both cell types converge around 1.4 Hz, with only negligible differences between MCF-10 and MCF-7. This suggests that despite structural and mechanical differences between normal and cancer cells, their overall dynamic stiffness (in terms of first natural frequency) is relatively close. Since the plateau is reached well before 400,000 elements, the results confirm that the model is mesh-independent beyond a certain level of discretization. Using excessively fine meshes would therefore increase computational cost without significantly improving accuracy.

### 3.4 Nonlinear differential equation result

**Fig. 7:**
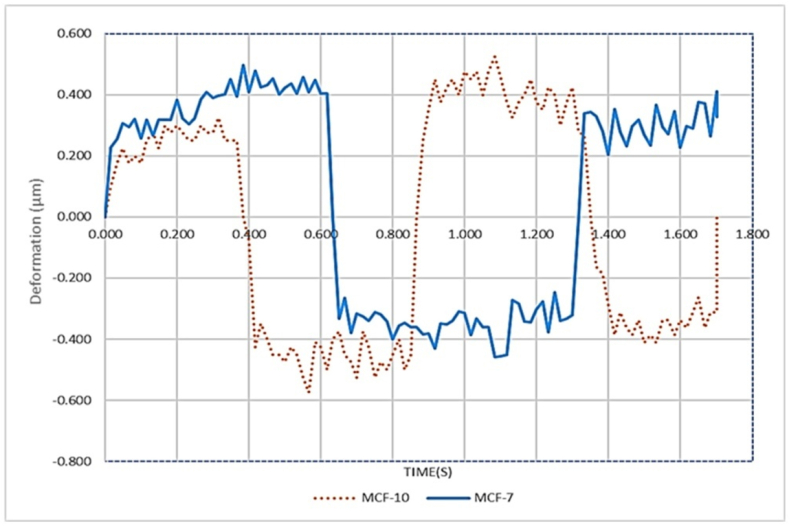
Time deformation of MCF10 (red dotted line) and MCF7 (blue solid line) cells over a 1.8 s interval, showing the greater amplitude fluctuations and irregular oscillations of the softer, malignant MCF7 cells compared with the more stable cycles of the stiffer, normal MCF10 cells. Chaos was analyzed across each frequency range, and the LLE was determined for each range (Table 2).

By applying a sinusoidal force and obtaining nonlinear data, followed by fitting the data with the Duffing model, the coefficients of the nonlinear Duffing model were determined. For the MCF-10 cells, the constants α and β that define the linear and nonlinear stiffness were 95.5592 *pN/m* and 12.6050 *pN/m*, respectively, while the damping coefficient δ was 2.0545 *pN s/m*. Similarly, for the MCF-7 cells, the coefficients were found to be α = 711.0313 *pN/m*, β = 4.1231 *pN/m*, and δ = 16.6562 *pN s/m* (Fig. 7). Fig. 7 illustrates how two different cell types (MCF10 healthy breast epithelial cells, shown as red dotted lines, and MCF7 breast cancer cells, shown as blue solid lines) respond to cyclic loading over time. Both groups display alternating periods of positive and negative deformation, reflecting repeated loading–unloading cycles, but they differ in the size and pattern of their responses, revealing variations in their mechanical properties.

For MCF-7 cancer cells, deformation increases steadily in the early stage (0–0.6 s) to about +0.4 μm, then shifts sharply into a negative range between 0.6 and 1.4 s, reaching −0.4 to −0.5 μm. This indicates high compliance and adaptability under cyclical forces. By the later stage (1.4–1.8 s), deformation recovers into the positive range, stabilizing near +0.3 to +0.4 μm. The smooth, large-amplitude oscillations suggest that these cancer cells are softer and more deformable, showing greater elasticity and plasticity.

MCF-10 normal cells begin with a moderate increase in deformation in the first 0.4 s, peaking around +0.25 μm. They then transition sharply to negative deformation between 0.4 and 1.2 s, down to roughly −0.6 μm, but with less stability than MCF-7. In the later stage (1.2–1.8 s), they return to the positive range near +0.3 μm, though with greater fluctuation and lower amplitude than the cancer cells. This indicates that normal cells are stiffer and less compliant, resisting larger shape changes under cyclic loads.

Overall, MCF-7 cells exhibit higher deformation amplitudes, smoother and more consistent oscillations, and greater softness than MCF-10 cells. These traits align with the known tendency of cancer cells to be mechanically more pliable, a property that aids their migration, invasion, and metastasis.

**Fig. 8:**
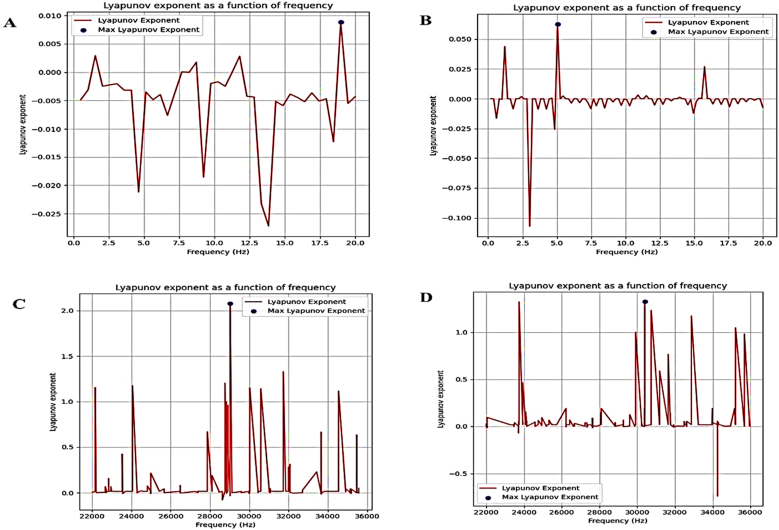
Chaos in Cell Lines Across Frequency Intervals, the blue dot represents the LLE in each graph: (0–20 Hz and 108,000–360,000 Hz), (A) 0–20 Hz MCF-10 Frequency of LLE: 18.97436 Hz, (B) 0–20 Hz MCF-7 Frequency of LLE: 5 Hz, (C) 22–36 kHz MCF-10 Frequency of LLE 29.0268 kHz, (D) 22–36 kHz MCF-7 Frequency of LLE: 30.3996 kHz.

The LLE diagram and LLE for the frequency range 0–20 Hz and 22–36 kHz are displayed for each cell group (Fig. 8).

In Panel A, which represents MCF-10 healthy breast epithelial cells at low frequencies (0–20 Hz), the Lyapunov exponent hovers around zero with mostly negative values, indicating overall stability. A small positive peak of about 0.01 appears near 19 Hz, suggesting only weak chaotic behavior at that upper end of the range.

Panel B shows MCF-7 breast cancer cells over the same low-frequency range. Here, the oscillations are stronger, with both negative and positive peaks. A clear maximum of about 0.055 at roughly 6 Hz points to a local instability and a sharper, more frequency-selective chaotic-like response than in MCF-10.

In the ultrasonic range (22–36 kHz), Panel C for MCF-10 displays multiple exponent spikes, the largest at about 2.0 near 28.5 kHz. This strong positive value reflects pronounced chaotic or unstable behavior, and the frequent positive values suggest that high-frequency acoustic stimulation drives substantial nonlinear effects.

Panel D, for MCF-7 in the same ultrasonic range, also shows several peaks, with the dominant one around 1.3 near 30 kHz. While the amplitude of instability is lower than that of MCF-10 in Panel C, it still represents significant sensitivity to high-frequency excitation.

In essence, both MCF-10 and MCF-7 are frequency-sensitive nonlinear systems. They remain mostly stable at low frequencies but can display chaotic dynamics at particular resonances roughly 19 Hz for MCF-10, about 6 Hz for MCF-7, and strong ultrasonic peaks for both. The difference lies in where those peaks occur and their intensity, with MCF-10 showing higher chaotic amplification at 28.5 kHz and MCF-7 peaking slightly later and at a lower amplitude.

## 4 Discussion

In Fig. 3, the dynamic creep response indicates that cancerous cells undergo more deformation than healthy cells. This result is consistent with another research. MCF-10 cells exhibit greater stiffness compared to MCF-7 cells, which aligns with previous studies. For instance, Milani et al. calculated the elastic modulus to be 237.25 and 715.71 N/m^2^ for MCF-7 and MCF-10, respectively [38].

The present study set out to examine the mechanical and dynamic behavior of normal MCF-10 breast epithelial cells and malignant MCF-7 breast cancer cells when exposed to various physical stimuli, integrating experimental force–indentation measurements, finite element simulations, frequency analysis, and nonlinear dynamic assessments. Force–indentation curves revealed distinct approach and retract patterns in both cell types, consistent with viscoelasticity, but MCF-7 cells displayed greater deformation under equivalent forces, indicating a softer cytoskeletal structure (Fig. 4- A). This was corroborated by finite element models, which showed that stress and displacement distributions were more concentrated in MCF-7, supporting their reduced stiffness and increased deformability (Fig. 4- B,C). Contour plots of displacement under mechanical loading demonstrated narrower, more uniform patterns in MCF-10, consistent with structural integrity and resistance to deformation, whereas MCF-7 exhibited broader, irregular patterns indicative of cytoskeletal weakening and diminished mechanical stability like features that promote migration and invasion in cancer (Fig. 3).

Nanomechanical measurements showed that many cancer cell lines, including breast cancer cells, exhibited reduced stiffness and altered actin architecture compared to non-transformed epithelial cells [39]. These changes were associated with increased deformability and altered frequency-dependent mechanical responses. Complementary modal and harmonic analyses of MCF-7 and MCF-10 cells also revealed differences in subcellular mechanical behavior and resonance, attributable to distinct cytoskeletal arrangements and effective stiffness, resembling wave responses [40].Also high-frequency electromagnetic can affect cytoskeleton and membrane. For instance, Heydarian et al. demonstrated that ionizing radiation alters the stiffness and elongation of healthy and cancer cells, effects that might arise through cytoskeletal breakdown and membrane polymerization [36]. These aspects should be investigated more in future studies.

Mesh-convergence analysis confirmed the reliability of the finite element approach, showing both cell types with stable natural frequency values near 1.4 Hz, suggesting that resonance-based detection would require finer resolution or supplementary parameters to discriminate between healthy and cancerous cells (Fig. 4). Time-dependent deformation showed both cell types undergoing periodic oscillations, though MCF-7 exhibited larger amplitude fluctuations and greater instability, while MCF-10 maintained more regular cycles, reflecting higher mechanical resilience in the normal cells (Fig. 5). Nonlinear dynamic analysis using Lyapunov exponents revealed that, in the 0–20 Hz range, MCF-7 had a distinct instability with a positive peak around 6 Hz, while MCF-10 remained stable except for a small chaotic peak near 19 Hz (Fig. 6). In the ultrasonic range (22–36 kHz), both entered chaotic regimes; however, MCF-10 exhibited a stronger response, with a maximum of approximately 2.0 at 28.5 kHz compared to MCF-7's peak of about 1.3 at 30 kHz. Collectively, the findings show that MCF-7 cells are softer, more deformable, and dynamically unstable at low frequencies, whereas MCF-10 cells are stiffer and more mechanically stable under such conditions but prone to pronounced chaotic responses under high-frequency ultrasound, highlighting the potential of combining low-frequency and ultrasonic stimulation for biomechanical differentiation between healthy and malignant cells.

Anggayasti et al. demonstrated that brief exposure to low-frequency mechanical vibrations (20 Hz for 1 h) significantly induces apoptosis in A431 epidermoid carcinoma cells without causing necrosis or harming surrounding healthy tissue [41]. In another study, researchers found that frequencies around 20–40 Hz are particularly effective for promoting osteogenesis and reducing cancer cell invasion [18]. Our FEM natural frequencies for both material models are in the same range as those found in other studies. However, since the resonant frequencies for healthy and cancerous cells are very close, the applied vibration could potentially destroy both types of cell structures. Mubeen et al. modeled both types of cells as elastic spheres containing a nucleus and cytoplasm. Their simulated natural frequencies for MCF-10 and MCF-7 cells were close, but not identical, as shown in their results (eg, 22,942 Hz vs. 9611 Hz). The discrepancy between their findings and ours is likely due to differences in model geometry and the number of layers used [42].

In addition, frequencies below 100 Hz, such as 40 Hz and 80 Hz, are notable for our nonlinear model. However, the LLE is not sufficient to lead the model to chaos. The natural frequencies of FEM are near 20 Hz and 40 Hz, but the natural frequencies of MCF-7 and MCF-10 are not distinguishable.

Yi et al. study showed that low-magnitude, high-frequency vibrations (90 Hz) applied to breast cancer cells significantly reduced their invasive capabilities by altering the secretion of osteolytic factors such as PTHLH, IL-11, and RANKL [43].

S.K. Jaganathan et al. also examined the natural frequencies of normal and cancer cells in the context of breast (MCF-10, MCF-7) and prostate cancer (BPH, LNCap) using elastic modeling and FEM with the assumption of spherical geometry for cells. Their results differ because changes in geometry alter the natural frequency [44]. For higher frequencies, A. Geltmeier et al. examined the dynamic behavior of MCF-7 and MCF-10 cells under low-frequency ultrasound using modal and harmonic analyses. The FEM simulated the mechanical properties of the cells, revealing resonance frequencies of approximately 21 kHz for MCF-7 cells and 34 kHz for MCF-10 cells. The study found that ultrasonic treatment at around 24.5 kHz reduced cell viability in MCF-7 cells, while frequencies above 29 kHz had a cytotoxic effect on MCF-10 cells [40]. In contrast to our study, we did not find any natural frequency for MCF-7 above 21 kHz, but according to our FE models, 26–27 kHz can be critical for MCF-10. In our nonlinear model, we found 29.0268 kHz and 30.3996 kHz to be chaotic frequencies for MCF-10 and MCF-7, respectively.

Because the nucleus plays a critical role in sensing mechanical signals, disruption of nuclear connectivity impaired the effectiveness of vibration in suppressing invasion and secretion of osteolytic factors. Conditioned media from mechanically stimulated cancer cells showed a substantial reduction in osteoclast activity with a significant reduction in osteoclast resorption capacity. Mechanical stimulation also altered gene expression related to cell adhesion and proliferation. This research highlights the potential of mechanical forces to influence breast cancer progression and inform therapeutic strategies targeting metastasis [43]. Based on this evidence, future work must employ a more comprehensive model that incorporates realistic cellular geometry and physiological conditions. Fraldi et al. demonstrated that the observed differences in stiffness between healthy and cancer cells allow their differentiation. They pointed out that the resonant frequencies (ranging from tens to hundreds of kilohertz) that dominate temperature fluctuations could be used to selectively target and destroy cancer cells [45]. This approach is supported by the experimental work of Heyden and Ortiz, who developed a technique called “oncotripsy” that uses ultrasonic harmonic excitation tuned to the specific resonant frequency of cancer cells [46]. In another line of research, the researchers used vibrating cantilevers to address the long-standing question of cellular natural frequencies, a challenge that has persisted for more than 70 years [47].

Collectively, these efforts are pioneering a new frontier in noninvasive, mechanistically based cancer treatment. However, cells, like other complex systems, are difficult to characterize. This study introduces a new paradigm by focusing on nonlinearity, identifying distinct bands of natural frequencies that could lead to the development of therapeutic devices. In addition, it lays the groundwork for future lab-on-a-chip technologies designed for natural frequency detection.

## 5 Conclusion

In this study, we investigated the effects of mechanical vibration on MCF-10 and MCF-7 cell lines both experimentally and through modeling. The natural frequencies obtained from the finite element method are close for both cell groups when using two stiffness models at lower frequencies. However, at higher frequencies, they are distinguishable. Identifying the natural frequency of cells can aid in devising devices to destroy cancer cells or utilize vibrated particle activation, though it depends on several factors such as geometry and stiffness. For further research, we recommend modeling cells with more accurate models. We utilized a nonlinear model and identified the frequencies that push the mechanical model to the edge of chaos. This method also has challenges, such as numerical calculation steps and the models used. These studies can serve as a fundamental basis for future research that can refine models and conduct cellular, animal, and human tests of mechanical dynamic force to treat cancers in a safe, non-thermal, and non-invasive way. Previous studies did not use similar approaches or cell lines, but we attempted to compare our results with other cancerous cell types.

This study highlights the crucial biomechanical differences between normal MCF-10 and cancerous MCF-7 cells using a combination of experimental indentation tests, finite element simulations, and nonlinear dynamic analyses. The results consistently demonstrated that MCF-7 cells are softer, more deformable, and dynamically less stable at low frequencies, whereas MCF-10 cells exhibit greater stiffness and mechanical resilience, but become more prone to chaotic responses at ultrasonic excitations. These findings not only confirm the role of cytoskeletal alterations in cancer cell mechanics but also suggest that frequency-dependent mechanical signatures could be exploited as diagnostic biomarkers. Specifically, the sensitivity of MCF-7 cells to low-frequency perturbations and the stronger chaotic response of MCF-10 under high-frequency excitation provide a potential basis for the development of non-invasive diagnostic or therapeutic strategies. Overall, integrating mechanical, dynamic, and nonlinear analyses offers a powerful framework for distinguishing healthy from malignant cells, and could open new avenues for biomechanical cancer research, early diagnosis, and mechanobiology-informed therapies.

In the end, we add some limitations and suggestions for future studies. One of the most important experiments that could complement the current study is the direct investigation of the effect of mechanical vibration on cytoskeleton. Live-cell imaging of F-actin and vinculin during high-frequency stimulation is needed to causally link cytoskeletal disruption with the chaotic thresholds reported here, and this can be pursued in future efforts. In the FEM simulation, we only reviewed a single three-dimensional model cell; this approach can be extended to multi-cell interactions and to developing a more comprehensive cell model that includes the nucleus, cytoskeleton, and other organelles.

## Funding

The authors received no financial support for the research, authorship, and/or publication of this article.

## CRediT authorship contribution statement

**Ashkan Heydarian:** Conceptualization, Project administration, Supervision. **Dornaz Milani:** Data curation, Methodology, Software. **Hamidreza Mortazavy Beni:** Software, Validation, Writing – review & editing. **Mehrafarin Babaee:** Visualization, Writing – original draft. **Hamid Reza Goudarzi:** Formal analysis, Validation, Writing – review & editing.

## Declaration of competing interest

The authors declare they have no conflict of interests.

## Data Availability

No data was used for the research described in the article.
